# The Effect of Complete Dentures on the Quality of Life of Edentulous Patients in the South Indian Population Based on Gender and Systemic Disease

**DOI:** 10.7759/cureus.4916

**Published:** 2019-06-17

**Authors:** Madhan K Seenivasan, Fathima Banu, Athiban Inbarajan, Parthasarathy Natarajan, Shanmuganathan Natarajan, V Anand Kumar

**Affiliations:** 1 Prosthodontics, Sri Ramachandra University, Chennai, IND; 2 Prosthodontics, Faculty of Dental Sciences, Sri Ramachandra Institute of Higher Education and Research, Chennai, IND

**Keywords:** quality of life, psychological and social comfort, standardized questionnaire, functional limitation, patient adaptation, rehabilitation

## Abstract

Purpose

Different socio-demographic variables, such as age, gender, and systemic disease, may affect satisfaction with complete dentures. Several studies have failed to show strong correlations either between patient satisfaction with their dentures and their quality or between denture satisfaction and the quality of the denture-supporting tissues. Hence, this study utilized a standardized questionnaire that included questions from domains such as mastication, appearance, speech, comfort, health, denture care, and social status. These questionnaires were used to determine the level of complete denture satisfaction along with socio-demographic variables such as age, gender, and systemic condition.

Materials and method

A total number of 128 completely edentulous patients aged between 40 and 50 years were selected. A standardized questionnaire, with 19 questions based on denture satisfaction level and masticatory capacity in the domains of functional limitation (FL), psychological discomfort (D1), psychological disability (D2), and social disability (D3), was administered. All the questions were recorded on a scale of 2, 1, 0 based on satisfied, moderately satisfied, and not satisfied, whereas hardly ever, occasionally, and very often were used for masticatory capacity. Questions on denture satisfaction were asked based on the post-treatment satisfaction with the new maxillary/mandibular complete dentures of the patients.

Results

Based on gender, the distribution of samples was 46.09% for male patients and 53.91% for female patients among the 128 patients selected. Similarly, based on systemic diseases, 66.41% had the presence of systemic disease while 28.13% did not have any systemic disease. Around 5.47% of the sample did not have any medical records. The predominance of psychological satisfaction was more for female patients. Based on systemic disease, it was observed that patients with the presence of systemic disease (Pn) were more psychologically comfortable than those who did not have systemic disease. The predominance of functional satisfaction was not marked in both genders. Based on systemic disease, it was observed that patients with the presence of systemic disease (Pn) had less functional comfort on mastication than those who did not have a systemic disease.

Conclusion

The acceptance of and satisfaction with complete denture treatment were comparatively higher in patients who had a systemic disease than in those with a non-systemic disease in terms of psychological and social comfort, whereas, in functionality, patients with a non-systemic disease had a higher satisfaction level.

## Introduction

Improved quality of life (QoL), together with a decline in mortality rates, has led to the growth of the elderly population worldwide. Several studies have failed to show strong correlations between either patient satisfaction with their dentures and their quality or denture satisfaction and the quality of the denture-supporting tissues [1-3]. Despite a global decrease in the edentulous rate, with great numbers of people reaching an advanced age, the number of patients without teeth continues to be high [4-5]. In India, being a developing country and having a huge population, there is a lack of awareness and management of the edentulous state and the rehabilitation of edentulous patients with complete dentures [6].

Since they are rarely life-threatening, little attention has been paid to the psychosocial impacts of oral conditions. Moreover, many researchers used to ignore the effects of the oral cavity on general health status, however, the need for the consideration of the oral health-related quality of life (QoL) has been increasingly acknowledged over the last decades and many studies highlight the psychosocial impacts of oral conditions. Edentulism is a chronic disease so functional improvement is more important than cure. The patient’s perception of the subjective experience of their denture is also important for dentists to motivate complete denture wearers, which is important for successful treatment.

Among the most important goals of dental care is helping patients in their attempts to reach an acceptable level of satisfaction with their oral cavity and dentition [7]. The literature contains many studies exploring the unique and vague relationship between psychological profiles and satisfaction with the dental status in many fields of dentistry [8]. Different socio-demographic variables, such as age, gender, and systemic disease, which may affect the satisfaction with complete dentures. Several studies have failed to show strong correlations between either patient satisfaction with their dentures and their quality or denture satisfaction and the quality of the denture-supporting tissues. Hence, this study utilized a standardized questionnaire that included questions from domains such as mastication, appearance, speech, comfort, health, denture care, and social status. These questionnaires were used to determine the level of complete denture satisfaction with socio-demographic variables such as age, gender, and systemic condition.

## Materials and methods

A total number of 128 completely edentulous patients who satisfied criteria, such as no past medical history, which affects the oral condition, new denture wearers, period of edentulousness varying between six months to one year, and a Class I completely edentulous state, as classified by the American College of Prosthodontics, were included in the study. Ethical clearance was obtained from the institution and subjects were voluntarily involved in the study. The subjects were in the age group of 40-50 years and grouped based on sex and on the presence or absence of systemic disease. Complete removable prostheses were fabricated and their quality was assessed based on the method given by Sato et al. [9]. The patients were interviewed at two to three months post-treatment. A single interviewer had conducted all the interviews to minimize variability. A standardized questionnaire, with 19 questions based on denture satisfaction level and masticatory capacity in the domains of functional limitation (FL), psychological discomfort (D1), psychological disability (D2), and social disability (D3), was administered (Table 1). All the questions were recorded on a scale of 2, 1, 0 based on satisfied, moderately satisfied, and not satisfied, whereas hardly ever, occasionally, and very often were used for masticatory capacity. Questions on denture satisfaction were asked based on the post-treatment satisfaction with the new maxillary/mandibular complete dentures of the patients. Statistical analysis was done using Statistical Package for Sciences (version 21.0, IBM Corp., Armonk, NY, US). The significance of the percentage error of the two groups was tested by the student t-test and p-value denoted the level of significance (p<.05).

**Table 1:** Questionnaire for evaluating satisfaction level and masticatory ability SAQ - Satisfaction level questionnaire, MCQ - Masticatory ability questionnaire

S.No	Questionnaire
SAQ 1	With respect to how comfortable your denture are, how satisfied are you
SAQ 2	With respect to being self-assured and self-conscious, how satisfied are you with your dentures
SAQ 3	Are you satisfied with your smile
SAQ 4	With respect to appearance, how satisfied are you
SAQ 5	with respect to your professional performance, how satisfied are you
SAQ 6	How do you feel pleasure you get from food, compared to your natural teeth
SAQ 7	With respect to chewing, how satisfied are you with your dentures
MCQ 1	In comparison with other people, do you perceive that you take longer to chew the foods during meals
MCQ 2	Have you been irritable when having meals with other people
MCQ 3	Do you feel uneasy during meals due to lack of denture security and instability
MCQ 4	have you been embarrassed when eating with other people in meals
MCQ 5	have you been totally unable to function because of problem with your denture
MCQ 6	Have you ever had to interrupt meals because of problem with your denture
MCQ 7	have you found it difficult to chew any foods because of problems with your denture
MCQ 8	Do you need any special food preparation to enable e=chewing (such as cooking cutting into small parts? humidification)
MCQ 9	How stable is your denture when eating food of certain consistency
MCQ 10	Do you need force to swallow foods after chewing
MCQ 11	Do you think your swallowing larger pieces of food due to lack of proper fragmentation
MCQ 12	Have you found it uncomfortable to chew any foods with dentures

## Results

Distribution of sample

Based on gender, among the 128 selected patients, 46.09% were male patients and 53.91% were female. Similarly, based on systemic diseases, 66.41% among the selected patients had the presence of a systemic disease while 28.13% did not have any systemic disease and around 5.47% of the patients did not have any medical records to know their medical conditions.

Psychological discomfort

On postoperative assessment, both male and female edentulous patients were well-satisfied with the prosthesis and were psychologically comfortable. Though there was no statistical difference between male (Mn) and female (Fn) patients, it was observed that the distribution of the sample was Mn = 36 and Fn = 37 for SAQ1, Mn = 37 and Fn = 40 for SAQ2, and Mn = 47 and Fn =52 for SAQ3. The predominance of psychological satisfaction was more for female patients numerically. Based on systemic disease, it was observed that patients with the presence of a systemic disease (Pn) were more psychologically comfortable than those who did not have any systemic disease (An) with Pn = 67 and An = 1 for SAQ1, Pn = 70 and An = 1 for SAQ2, and Pn = 80 and An = 10 for SAQ (Table 2).

**Table 2:** Psycological discomfort based on gender SAQ - Satisfaction level questionnaire, MCQ - Masticatory ability questionnaire

Questionnaire	Gender	Satisfied	Moderately Satisfied	Not Satisfied	Pearson Chi-Square P-value
SAQ4	Male	36	14	8	0.54707
	Female	37	18	14	
SAQ5	Male	37	15	7	0.31772
	Female	40	14	15	
SAQ9	Male	47	7	5	0.25879
	Female	52	5	12	
MCQ9	Male	36	23	0	0.3721
	Female	38	29	2	
MCQ12	Male	52	7	0	0.00659
	Female	45	20	4	

Based on masticatory ability, both male and female edentulous patients had improved psychological comfort on mastication with the prosthesis. Though there was no statistical difference between male and female patients, it was observed that the distribution of the sample was Mn = 36 and Fn = 38 for MCQ1 and Mn = 52 and Fn =45 for MCQ2. It was also found that the predominance of psychological satisfaction was more for female patients numerically. Based on systemic disease, it was observed that patients with the presence of a systemic disease (Pn) were more satisfied with masticatory ability than those who did not have any systemic disease (An) with Pn = 67 and An = 1 for MCQ1, Pn = 85 and An = 1 for MCQ2 (Table 3).

**Table 3:** Psycological discomfort based on systemic diseases SAQ - Satisfaction level questionnaire, MCQ - Masticatory ability questionnaire

Questionnaire	Systemic Disease	Satisfied	Moderately Satisfied	Not Satisfied	Pearson Chi-Square P value
SAQ 4	No record	1	1	1	0.59798
	Present	67	0	18	
	Absent	1	27	1	
SAQ 5	No record	1	1	1	0.57793
	Present	70	1	5	
	Absent	1	20	16	
SAQ 9	No record	2	0	1	0.40062
	Present	80	0	1	
	Absent	10	10	13	
MCQ 9	No record	2	1	0	0.99377
	Present	67	1	2	
	Absent	1	44	0	
MCQ 12	No record	3	0	0	0.73416
	Present	85	1	0	
	Absent	1	24	4	

Social disability

On postoperative assessment, both male and female edentulous patients were well-satisfied with their social ability after wearing the prosthesis. Though there was no statistical difference between male and female patients, it was observed that the distribution of samples was Mn = 45 and Fn = 41 for SAQ4 and Mn = 42 and Fn = 44 for SAQ5. The predominance of psychological satisfaction was not well-marked in both genders. Based on systemic disease, it was observed that patients with the presence of systemic disease (Pn) were more socially comfortable than those who did not have systemic disease (An), with Pn= 35 and An = 38 for SAQ4 and Pn = 78 and An = 1 for SAQ4 (Table 4).

**Table 4:** Social disability based on gender SAQ - Satisfaction level questionnaire, MCQ - Masticatory ability questionnaire

Questionnaire	Gender	Satisfied	Moderately Satisfied	Not Satisfied	Pearson Chi-Square P-value
SAQ3	Male	45	8	6	0.12622
	Female	41	15	13	
SAQ7	Male	42	9	8	0.66002
	Female	44	14	11	
MCQ10	Male	40	19	0	0.29288
	Female	50	17	2	
MCQ11	Male	50	9	0	0.05624
	Female	48	17	4	
MCQ13	Male	46	12	1	0.4096
	Female	48	16	4	

Based on masticatory ability, both male and female edentulous patients had improved social ability on mastication with a prosthesis. Though there was no statistical difference between male and female patients, it was observed that the distribution of samples was Mn = 40 and Fn = 50 for MCQ3, Mn = 50 and Fn =48 for MCQ4, and Mn = 46 and Fn = 48 for MCQ5. Based on systemic disease, it was observed that patients with the presence of systemic disease (Pn) had more masticatory ability based on social ability than those who did not have systemic disease (An) with Pn= 80 and An = 1 for MCQ3, Pn = 85 and An = 1 for MCQ4, and Pn = 86 and An = 1 for MCQ5 (Table 5).

**Table 5:** Social disability based on systemic diseases SAQ - Satisfaction level questionnaire, MCQ - Masticatory ability questionnaire

Questionnaire	Systemic Disease	Satisfied	Moderately Satisfied	Not Satisfied	Pearson Chi-Square P value
SAQ 3	No record	1	1	1	0.46574
	Present	78	0	1	
	Absent	1	17	18	
SAQ 7	No record	1	0	2	0.09377
	Present	78	2	1	
	Absent	1	19	16	
MCQ 10	No record	2	1	0	0.96253
	Present	80	31	2	
	Absent	1	1	0	
MCQ 11	No record	2	1	0	0.71968
	Present	90	19	4	
	Absent	1	1	0	
MCQ 13	No record	3	0	0	0.69554
	Present	86	21	5	
	Absent	1	1	0	

Functional limitation

On postoperative assessment, both male and female edentulous patients were functionally well-satisfied on wearing the prosthesis. Though there was no statistical difference between male and female patients, it was observed that the distribution of samples was Mn = 35 and Fn =38 for SAQ6 and Mn = 40 and Fn = 35 for SAQ7. The predominance of functional satisfaction was not well-marked in both genders. Based on systemic disease, it was observed that patients with the presence of systemic disease (Pn) had less functional comfort on mastication than those who did not have systemic disease (An), with Pn = 13 and An = 55 for SAQ6 and Pn= 14 and An = 56 for SAQ7 (Table 6).

**Table 6:** Functional limitation based on systemic disease SAQ - Satisfaction level questionnaire, MCQ - Masticatory ability questionnaire

Questionnaire	Systemic Disease	Satisfied	Moderately Satisfied	Not Satisfied	Pearson Chi-Square P value
SAQ 1	No record	1	0	1	0.98279
	Present	13	5	5	
	Absent	55	19	21	
SAQ 2	No record	1	0	1	0.93491
	Present	14	5	4	
	Absent	56	24	15	
MCQ1	No record	1	1	0	0.90129
	Present	17	6	1	
	Absent	69	26	2	
MCQ 2	No record	0	1	1	0.81405
	Present	13	9	1	
	Absent	57	36	2	
MCQ 3	No record	1	1	1	0.39844
	Present	18	4	1	
	Absent	61	30	4	
MCQ 4	No record	1	1	1	0.52638
	Present	16	7	0	
	Absent	58	33	4	
MCQ 5	No record	1	1	1	0.68488
	Present	17	6	0	
	Absent	69	23	3	
MCQ 6	No record	1	1	1	0.80203
	Present	15	7	1	
	Absent	66	27	2	
MCQ 7	No record	1	1	1	0.65814
	Present	15	8	0	
	Absent	63	29	3	

Based on masticatory ability, both male and female edentulous patients had improved functional ability on mastication with a prosthesis. Though there was no statistical difference between male and female patients, it was observed that the distribution of samples was Mn = 45 and Fn = 43 for MCQ6, Mn = 35 and Fn = 38 for MCQ7, Mn = 41 and Fn = 42 for MCQ8, Mn = 37 and Fn = 41 for MCQ9, Mn = 43 and Fn = 49 for MCQ10, Mn = 40 and Fn = 47 for MCQ11, and Mn = 40 and Fn = 42 for MCQ12. Based on systemic disease, it was observed that patients with the presence of systemic disease (Pn) had less masticatory ability based on function than those who did not have systemic disease (An), with Mn = 17 and Fn = 69 for MCQ6, Mn = 13 and Fn = 57 for MCQ7, Mn = 18 and Fn = 61 for MCQ8, Mn = 16 and Fn = 58 for MCQ9, Mn = 17 and Fn = 69 for MCQ10, Mn = 15 and Fn = 66 for MCQ11, and Mn = 15 and Fn = 63 for MCQ12 (Table 7).

**Table 7:** Functional limitation based on gender SAQ - Satisfaction level questionnaire, MCQ - Masticatory ability questionnaire

Questionnaire	Gender	Satisfied	Moderately Satisfied	Not Satisfied	Pearson Chi-Square P-value
SAQ1	Male	35	13	11	0.71574
	Female	38	14	17	
SAQ2	Male	40	12	7	0.14162
	Female	35	20	14	
MCQ1	Male	45	14	0	0.08959
	Female	43	26	1	
MCQ2	Male	35	24	0	0.26369
	Female	38	28	3	
MCQ3	Male	41	16	2	0.59538
	Female	42	24	3	
MCQ4	Male	37	21	1	0.67826
	Female	41	25	3	
MCQ5	Male	43	15	1	0.89698
	Female	49	18	2	
MCQ6	Male	40	19	0	0.24671
	Female	47	19	3	
MCQ7	Male	40	19	0	0.23854
	Female	42	24	3	

## Discussion

Feine et al. reported that patient satisfaction with therapy is likely to be the distinguishing outcome of many treatments for chronic diseases for which living with treatment is a more realistic objective than cure [10].

Females with fitted, conventional, complete dentures reported less satisfaction with aesthetics and ability to chew than males [11]. Nevertheless, some studies reported no significant relationship between gender and satisfaction with complete denture treatment [12-15]. However, others found that males were more satisfied with dentures [16]. However, not all complete denture wearers are able to adapt to their dentures, even if the dentures fulfilled all conventional prosthodontic criteria.

Our findings concerning the qualities of stability, retention, occlusion, articulation, and vertical dimension of the complete dentures, together with the observation that these features become impaired with the increasing age of the dentures, support the findings of Hoad-Reddick (1989) [17].

A comparison of these features with the age of the dentures caused some problems, however, because a number of the elderly people had difficulty in recalling the history of their dental treatment. Of course, this may result from their advanced age and/or the prevalence of mild dementia diagnosed in the medical examination of these subjects, which ranged from 5% in the youngest age group to 27% in the oldest (Juva et al., 1993) [18].

The degree of satisfaction based on each of these elements for 120 patients (both males and females) were separately averaged, as presented in Table 2. Moreover, the degree of satisfaction based on the individual elements was found to impart no significant difference apart from that based on the function criterion where the males' degree of satisfaction was 86.36% while that of females was 65.8%. This may be due to the male patients generally giving more important to function, whereas for the female patients, aesthetics was a main concern.

Patient adaptation to the reestablishment varies as adaptation depends on neuromuscular control, but we were able to see that three months was sufficient for the majority of patients to achieve improvement in these symptoms. Several factors can influence satisfaction. They are interrelated and frequently have an associated effect. They include not only factors exclusive to the dental prosthesis, such as comfort, ability to masticate, aesthetics, and retention but the systemic health or general health of the patient also had an effect on adaptation.

Some of the systemic diseases that adversely affect patient’s satisfaction with their dentures include hyposalivation, Parkinson’s disease, myasthenia gravis, bulbar palsy, and diseases with either a strong connection to emotional stress or impairing mental health [19-20]. The ability to adapt to new dentures and the prognosis will generally diminish in proportion to the health status.

The effect of gender variation on patient’s satisfaction with their dentures has also been examined. It was found that men were generally more satisfied with their dentures when compared to women except when it came to aesthetics, where women scored higher. In this study, the treatment of rehabilitated patients with a total loss of teeth was analyzed in terms of patient QoL by administering the OHIP-EDENT (a specific questionnaire for edentulous patients (EDENT) based on the OHIP) before and three months after rehabilitation with a new complete denture.

## Conclusions

The acceptance and satisfaction of complete denture treatment were comparatively higher in patients with systemic disease than in patients with non-systemic disease in terms of psychological comfort. Female patients are more psychologically satisfied as compared to male patients.

In terms of social disability, patients with systemic disease are more socially comfortable than patients with a non-systemic disease. Again, females are more socially comfortable.

Based on functionality, patients with a non-systemic disease have a higher complete denture treatment satisfaction level and higher masticatory ability as compared with non-systemic disease patients.
